# Streambed microstructure predicts evolution of development and life history mode in the plethodontid salamander *Eurycea tynerensis*

**DOI:** 10.1186/1741-7007-4-6

**Published:** 2006-03-02

**Authors:** Ronald M Bonett, Paul T Chippindale

**Affiliations:** 1 Department of Biology, University of Texas at Arlington, Arlington TX 76019, USA; 2 Museum of Vertebrate Zoology and Department of Integrative Biology, University of California, Berkeley, CA 94720, USA

## Abstract

**Background:**

Habitat variation strongly influences the evolution of developmentally flexible traits, and may drive speciation and diversification. The plethodontid salamander *Eurycea tynerensis *is endemic to the geologically diverse Ozark Plateau of south-central North America, and comprises both strictly aquatic paedomorphic populations (achieving reproductive maturity while remaining in the larval form) and more terrestrial metamorphic populations. The switch between developmental modes has occurred many times, but populations typically exhibit a single life history mode. This unique system offers an opportunity to study the specific ecological circumstances under which alternate developmental and life history modes evolve. We use phylogenetic independent contrasts to test for relationships between a key microhabitat feature (streambed sediment) and this major life history polymorphism.

**Results:**

We find streambed microstructure (sediment particle size, type and degree of sorting) to be highly correlated with life-history mode. *Eurycea tynerensis *is paedomorphic in streams containing large chert gravel, but metamorphoses in nearby streams containing poorly sorted, clastic material such as sandstone or siltstone.

**Conclusion:**

Deposits of large chert gravel create loosely associated streambeds, which provide access to subsurface water during dry summer months. Conversely, streambeds composed of more densely packed sandstone and siltstone sediments leave no subterranean refuge when surface water dries, presumably necessitating metamorphosis and use of terrestrial habitats. This represents a clear example of the relationship between microhabitat structure and evolution of a major developmental and life history trait, and has broad implications for the role of localized ecological conditions on larger-scale evolutionary processes.

## Background

The ability to express alternate phenotypes enables populations to colonize and persist in variable environments [1-4]. However, the role of phenotypic plasticity in the evolution of traits, and subsequent diversification and speciation, is understood in relatively few instances [4]. Populations experiencing a given environmental regime across generations may undergo directional selection, from a highly plastic trait to a single condition [4-6], thus initiating phenotypic divergence among populations that occupy different environments. Furthermore, selection acting on reproductive traits may lead to reproductive isolation among populations. Especially for organisms with complex life cycles, local selection involving major developmental shifts can produce rapid changes in ecology, morphology and life history among populations, and may hasten divergence and speciation [7-9].

Amphibians exhibit diverse life history strategies that enable them to exploit both aquatic and terrestrial ecosystems successfully [10-13]. Most amphibians have a free-living aquatic larval stage followed by metamorphosis into a more terrestrial adult form [10-13]. One deviation from this pathway, exhibited by some salamanders, is paedomorphosis, whereby individuals reach adulthood while maintaining many larval structures (particularly gills) and an aquatic lifestyle [10,14]. In some groups of salamanders the switch between paedomorphosis and metamorphosis can be highly labile, and a multitude of ecological and evolutionary trade-offs accompany each strategy [extensively reviewed in [15-17]]. The most important advantage of metamorphosis is the ability to leave ephemeral aquatic habitats [11,15,18,19], and in some situations metamorphosis could potentially increase gene flow between otherwise isolated aquatic systems [20]. In permanently aquatic environments, especially those of high quality, paedomorphosis may be optimal [11,15,21-24]. Also, paedomorphs remain in their breeding habitat, limiting mortality from breeding migrations and providing the chronological advantage of early arrival [23,25]. In regions where peripheral habitats are extremely harsh or dry, remaining in the aquatic environment may be the only viable strategy [11,26-28].

The Ozark Plateau of south-central North America is a geologically diverse highland with many endemic species [29-31]. Watercourses carve through the plateau at several distinct stratigraphic levels, causing the deposition of different types of sediment among streams [32]. Silurian/Ordovician chert strata are very hard and deposit as loosely associated gravel in many western Ozark streams, whereas in most other localities stream sediment is composed of densely packed clastic sediments such as sandstone and siltstone. The interstitial spaces within chert gravel beds harbour a diverse fauna, including a variety of arthropods, small fishes, and salamanders. Streambed microstructure is critical for small aquatic organisms in these communities, because it provides pathways to underground water during harsh dry summer conditions, when the surface can dry completely [29,33-35].

The plethodontid salamander *Eurycea tynerensis *is restricted to the Ozark Plateau, and, until very recently, was considered to be a strictly paedomorphic species, with a distribution closely associated with Silurian/Ordovician chert gravel streams in the western Ozarks [33-36]. However, recent molecular phylogenetic evidence revealed that *E. tynerensis *is composed of paedomorphic and metamorphic populations (the latter formerly considered *E. multiplicata griseogaster*), and paedomorphosis appears to have arisen independently many times [37]. Populations of *E. tynerensis *characteristically exhibit a single life-history mode, suggesting that local habitat parameters are highly influential, via either selection for a facultative response or for particular genotypes [37]. This system offers a unique opportunity to investigate the role of local microhabitat conditions responsible for major phenotypic divergence.

**Figure 1:**
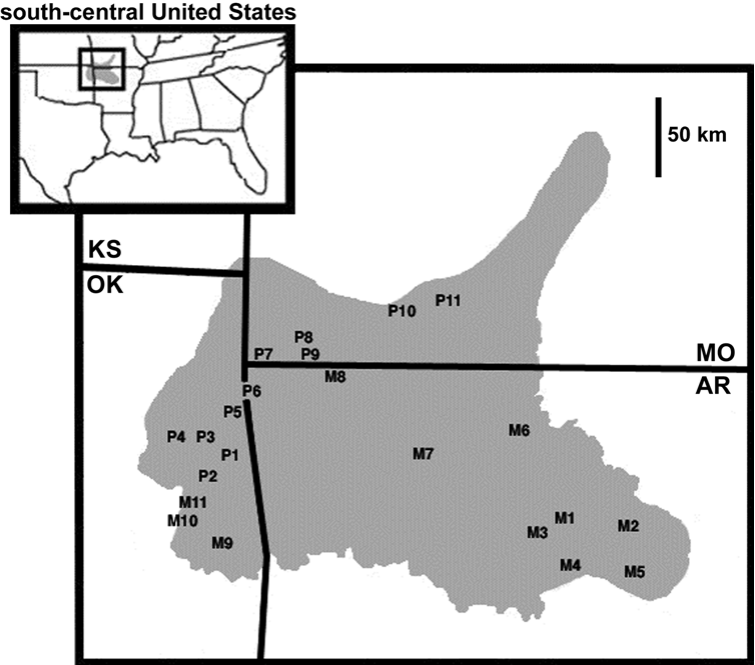
**Distribution of *E. tynerensis *and localities sampled**. Geographic distribution of *Eurycea tynerensis *(shaded) in the south-central United States. Locality codes correspond to those in Additional File 1, and indicate life history mode (M = metamorphic and P = paedomorphic). The states Arkansas, Kansas, Missouri and Oklahoma are abbreviated AR, KS, MO and OK, respectively.

Here we investigate the importance of streambed microstructure in the evolution of a major life-history polymorphism. We quantify substrate size, degree of sorting and type for 22 populations (11 paedomorphic and 11 metamorphic; Fig. 1) of *E. tynerensis *from across the Ozark Plateau. Using a phylogeny based on 1,818 bp of the mitochondrial genes *cob *and *nad4 *to assess relationships among populations, we test for correlations between substrate parameters (size, sorting and type) and life-history mode (paedomorphic vs. metamorphic) using phylogenetic independent contrasts. On the basis of our results we make inferences about the evolution and maintenance of alternate life-history modes in this group across the geologically diverse Ozark Plateau. Furthermore, we discuss the role of microhabitat in initiating local divergence, which ultimately may result in large-scale changes in patterns of organismal development, morphology, ecology and life history, as well as formation of new species.

## Results

### Sequence variation and phylogeny

**Figure 2:**
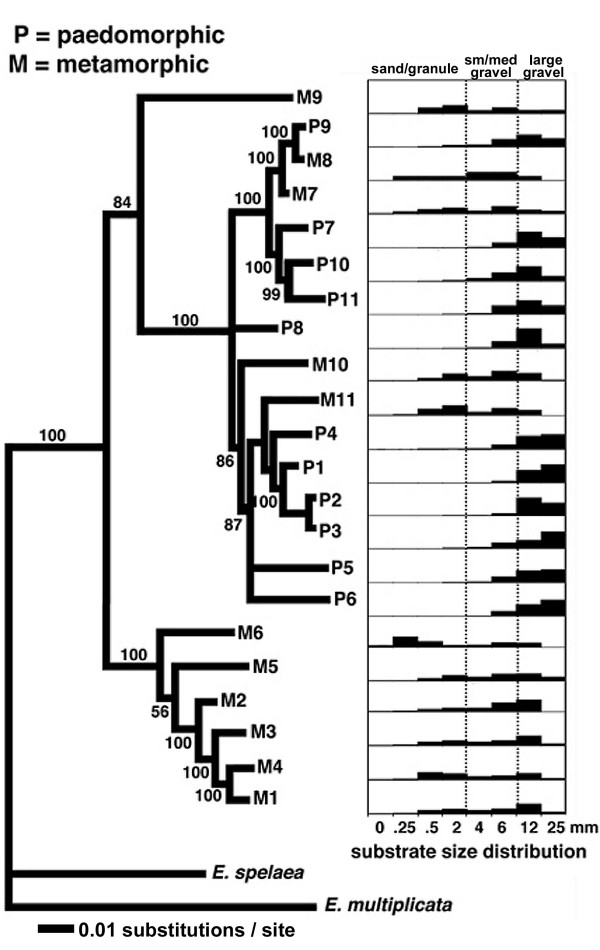
**Phylogeny and size distribution of substrate samples**. Bayesian phylogeny of 22 populations of *E. tynerensis*. Topology is based on a majority rule consensus of 50,000 trees (5 × 10^6 ^generations sampled every 100 generations). Branch lengths were averaged across 50,000 trees. Posterior probabilities indicate node support, and only those ≥ 50% are shown. Histograms show proportions of substrate in each size class for each locality. Boundaries of the three size categories: sand and granule (0 to <2 mm), small/medium gravel (>2 to < 12 mm) and large gravel (≥ 12 mm) are indicated with dotted lines.

The concatenated *cob *and *nad4 *sequences for all individuals formed an alignment of 1,818 homologous nucleotides. Each individual displayed a unique haplotype and uncorrected pairwise sequence divergence ranged from 0.22 to 9.35 % among the 22 populations of *E. tynerensis*. Bayesian analysis yielded a well-resolved tree with strong support for most relationships (Fig. 2). Maximum parsimony analysis and nonparametric bootstrapping (not shown) yielded very similar, well-supported trees. Phylogenetic independent contrasts calculated using the topology of the Bayesian tree were nearly identical to those based on the maximum parsimony topology. Given the similarity between the results of both methods, and since Bayesian analyses provide a well-parameterized, model-based estimation of branch lengths, we only show the results based on the Bayesian tree.

### Independent contrasts of substrate parameters

**Figure 3:**
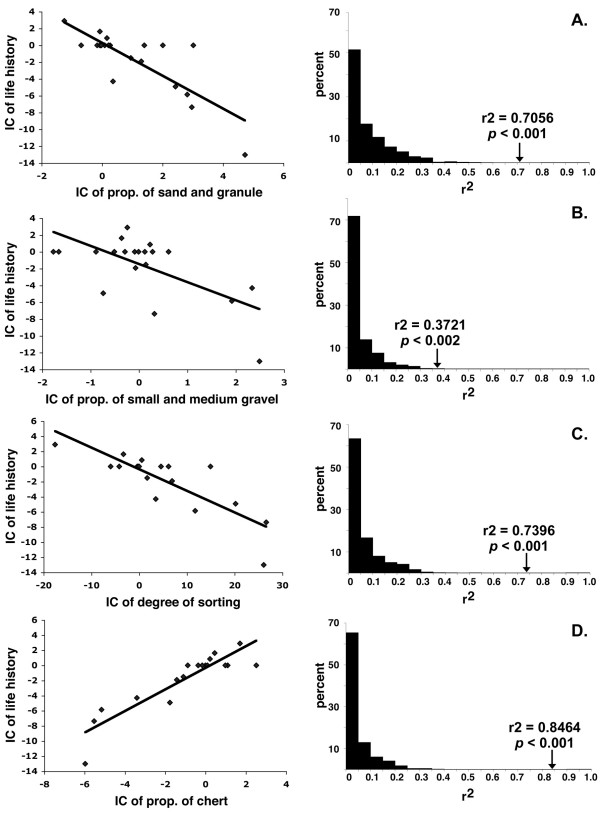
**Relationships of substrate parameters to life history mode**. Regression plots (left) of independent contrasts of substrate parameters (**A**, proportion of sand and granule; **B**, proportion of small/medium gravel; **C**, degree of sorting; **D**, proportion of chert) versus independent contrasts of life history mode. Histograms (right) represent the distribution of r^2 ^values based on 1000 MCMC randomizations of the original data. Arrows indicate location of observed r^2 ^relative to the randomized distribution.

The distribution of substrate size differed in metamorphic (M) versus paedomorphic (P) localities (Fig. 2). Most notably, P localities lack substrate of the smaller size classes. Phylogenetic independent contrasts (PICs) [38-40] are powerful methods that account for ancestry when testing for relationships between putative adaptations, or adaptations and the environment. For each locality we determined the proportion of sediment in three broad size categories: sand/granule (0 to < 2 mm), small/medium gravel (2 to < 12 mm), and large gravel (≥12 mm). Using PICs we found a significant negative correlation between paedomorphosis and the presence of material in the sand and granule (r^2 ^= 0.7056; *p *< 0.001; Fig. 3A) and small/medium gravel (r^2 ^= 0.3721; *p *< 0.002; Fig. 3B) categories.

Sorting is a measure of the uniformity of particle sizes in a substrate sample [41], and is an important parameter for determining overall streambed compactness (embeddedness [33,34]). Streambeds composed of well sorted large material provide open spaces between particles, whereas weakly sorted streambeds (containing multiple size classes) are much less penetrable because small material fills in any interstitial spaces (see also discussion of embeddedness below). To determine the degree of sorting, we counted the number of size classes (1 to 8) represented in the substrate samples at each locality. The size class had to represent at least 5% of the total substrate sample in order to be counted. The substrate in P localities is very well sorted (i.e. composed of few size classes), whereas M localities had substrate in many size classes. We found a strong negative correlation between the number of size classes and the presence of paedomorphs (r^2 ^= 0.7396; *p *< 0.001; Fig. 3C), indicating that substrate particles were well sorted in P and poorly sorted in M localities. Our samples were primarily composed of clastic rocks (sandstone and siltstone) and chert. Clastic rocks are composed of smaller particles (e.g., sand and silt), while chert is an extremely hard siliceous substance. *Eurycea tynerensis *previously was found to be closely associated with chert gravel, but transforming populations, which were thought to be a different species, were not examined in earlier studies [33-35]. Therefore, to quantify substrate type for both life history modes we measured the proportion of the volume of each sample that was composed of chert versus clastic material at each locality. The proportion of chert present in samples ranged from 0 to 97 %. Paedomorph localities were primarily composed of chert, while the M localities primarily contained clastic material. We found a strong positive correlation between the presence of chert and paedomorphosis (r^2 ^= 0.8464; *p *< 0.001; Fig. 3D). Conversely, metamorphosis was strongly correlated with clastic substrates.

## Discussion

### Streambed microstructure and life history of *E. tynerensis*

**Figure 4:**
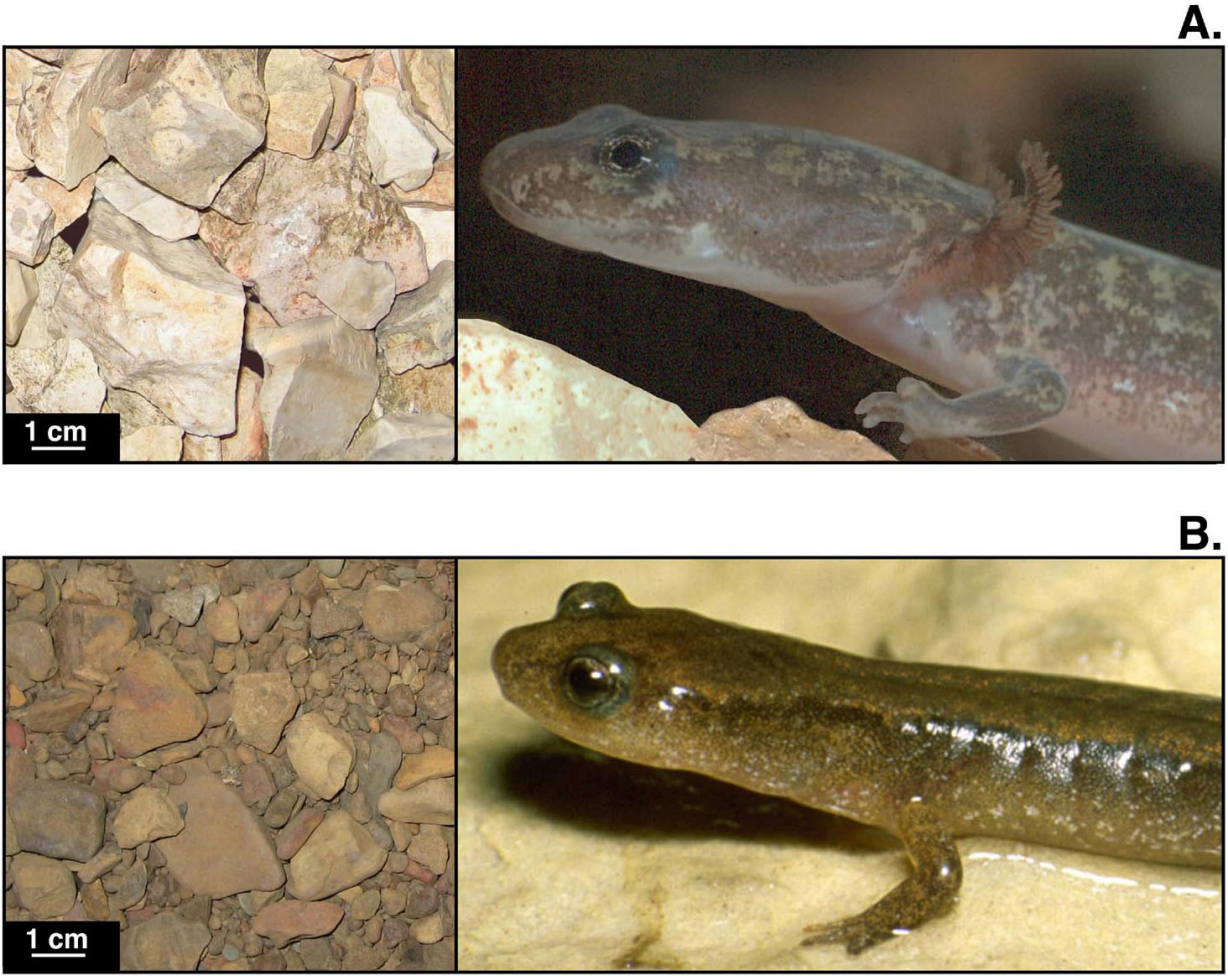
**Alternate life history modes of *E. tynerensis *and associated substrates. A**, Paedomorphic *Eurycea tynerensis *and an example of Silurian/Ordovician chert gravel from sites where paedomorphs occur. Note the large particle sizes that provide interstitial spaces in the substrate. **B**. Metamorphosed *E. tynerensis *and an example of densely packed and poorly sorted clastic rock typical of metamorphic habitat. Scale bars only apply to the substrate pictures. Salamander photographs compliments of R. W. Van Devender.

Previous work has shown the presence of *E. tynerensis *to be associated with Silurian and Ordovician chert gravel streams in the western Ozark Plateau [33-35]. However, because the authors of these studies considered only paedomorphs as *E. tynerensis*, they were studying not the distribution of the species but of a life history mode that we now know to be independently derived in different parts of a complex clade. In the light of new evidence that *E. tynerensis *comprises both paedomorphic and transforming populations [37], we found that this developmental and life history polymorphism is strongly correlated with several substrate parameters (size, degree of sorting and type). These variables create distinctly different streambed microstructures where *E. tynerensis *occurs (Fig. 4), which has major implications for the evolution of alternate life history modes.

Consistent with previous studies [33-35], we found that paedomorphic populations are primarily restricted to streams with chert gravel, which is very well sorted into large size classes. Various factors have been hypothesized to explain why chert gravel is important for paedomorphic *E. tynerensis *[33-35]. Chert is an extremely hard substance that breaks into sharp conchoidal patterns, producing highly irregular shapes. When chert gravel is deposited it creates very porous streambeds with large interstitial spaces that can be utilized by small aquatic organisms [33-35]. Previous studies have shown highly embedded streams (containing a high percentage of fine sediment) to be devoid of paedomorphic *E. tynerensis *[33,34]. Owing to the very hard nature of chert, fracturing is the primary mode of decomposition, and very little fine material is released during a break. Furthermore, the irregular shapes and sharp edges of chert have been proposed to promote turbulent currents between interstitial spaces, which further inhibit deposition of fine sediments [35]. In most localities the interstitial spaces between the chert gravel are crucial to the existence of paedomorphic *E. tynerensis *for avoidance of both predators and desiccation [33-35]. During xeric summer conditions, most small streams across the Ozark Plateau dry completely at the surface. Interstitial spaces provide pathways for small fishes, crayfish, aquatic salamanders and other aquatic organisms to access the water table far below the surface [29,34,35]. During extreme drought years, paedomorphic individuals of *E. tynerensis *have been found at depths as great as 2.5 meters below the surface [29].

We found the substrate parameters for metamorphic populations to be different from those for paedomorphic populations. In general, substrates in localities where *E. tynerensis *metamorphoses contain very little chert gravel and are primarily composed of poorly sorted and relatively smaller clastic (sandstone and siltstone) materials. Clastic rocks are formed from compression of particulate matter such as sand (sandstone) and silt (siltstone). Erosion of clastic rocks causes them to break down gradually into constituents. For sandstone and siltstone, this causes the deposition of a wide range of particle sizes (i.e., poorly sorted material [41]) in streams where these rocks occur. Small sediments fill in the interstitial spaces between larger particles, forming a densely packed streambed microstructure that is largely impenetrable [33]. Therefore the substrate blocks access to any potential subterranean water, so when surface water dries, metamorphosis is presumably critical at these localities. Metamorphic *E. tynerensis *have a relatively brief larval period (seven to eight months) that matches the seasonality of surface habitat moisture in the Ozark Plateau [42].

Owing to its direct connection with subsurface water, the microstructure of streambed sediments appears to be a major habitat feature determining life history mode in *E. tynerensis*, although it may not be the only factor. Nutrient abundance, persistence of underground water, availability of suitable peripheral habitat, or many other factors may also influence the distribution of alternate life history modes in this group. The terrestrial habitats surrounding paedomorphic *E. tynerensis *localities are often very dry, because porous chert streambeds that extend outside the stream proper are unable to sustain moisture. The lack of suitable peripheral habitat for metamorphosing salamanders probably favours selection for paedomorphosis, or the ability to shift facultatively to this life-history mode. A similar situation occurs in the hot, dry Edwards Plateau of central Texas: naturally metamorphosing populations of *Eurycea *(members of the *E. troglodytes *complex sensu [43,44]) appear limited to a small region of mesic canyons with moist streamside habitat, whereas all other known *Eurycea *in the region are strictly paedomorphic [44,45].

The relationships among all the 26 recognized species of *Eurycea *[46] are unclear, but at least one highly relevant aspect of the phylogeny has been established. *Eurycea tynerensis *is phylogenetically nested in a clade of otherwise strictly metamorphosing species (*E. multiplicata *and *E. spelaea*) [37,47]. Thus, paedomorphosis, or the ability to be facultatively paedomorphic, probably evolved independently in the ancestor of the *E. tynerensis *group, while the ability to metamorphose has also been maintained. Paedomorphosis enables the exploitation of unique chert gravel bottom streams that allow continuous access to permanent water, while metamorphosis has permitted the continued colonization of seasonally ephemeral aquatic habitats throughout the Ozark Plateau. Several other species of *Eurycea *(*E. longicauda*, *E. lucifuga*, and *E. spelaea*) are broadly sympatric with *E. tynerensis*, however none exhibit paedomorphosis [48]. This may explain their restriction to the headwaters of springs and to caves that contain water, whereas the distribution of *E. tynerensis *extends much further throughout the stream [49]. This further supports the hypothesis that some independently evolved feature of the *E. tynerensis *clade governs paedomorphosis.

Here we treat *E. tynerensis *as a single species, the most conservative approach given the well-supported monophyly of the group. However, especially given the levels of sequence divergence present, it seems likely that numerous independent lineages are evolving in response to readily quantifiable habitat parameters, and multiple species may already be present. Whether because phenotypic plasticity is retained over long periods of evolutionary time or metamorphosis versus paedomorphosis is repeatedly selected for, members of the *E. tynerensis *group represent a clear example of evolutionary developmental change in vertebrates attributable to differences in habitat microstructure.

### Developmental divergence in salamanders

Nine of the 10 salamander families exhibit paedomorphosis [10,14,48], a developmental deviation from the typically biphasic amphibian life cycle to a strictly aquatic one. All species in the families Amphiumidae, Cryptobranchidae, Proteidae, and Sirenidae are paedomorphic, whereas within five other families (Ambystomatidae, Dicamptodontidae, Hynobiidae, Plethodontidae, and Salamandridae), paedomorphosis is variable within and among species. All species of amphiumids, cryptobranchids, proteids, and sirenids, as well as some plethodontids and ambystomatids, are obligate paedomorphs (i.e., cannot metamorphose) in nature [10,12,14]. For many of these species the ability to metamorphose has been completely lost, and cannot even be induced experimentally with hormonal treatment [12,14]. Conversely, several species of ambystomatids, salamandrids, and possibly other salamanders are facultatively paedomorphic, with life-history mode dictated by environmental conditions [10]. This plasticity allows populations, and even individuals, to change strategies when faced with fluctuating environmental conditions. Individuals of some species may be paedomorphic for part of their life (i.e. reproduce in the aquatic larval form) prior to metamorphosis [48,50].

Selection for plasticity in life-history strategies allows individuals, populations and/or species to persist in unpredictable environments [7]. However, selection on populations in temporally very stable environments may favour a single mode of life history and eliminate another [21,23,51]. This seems to be the case for several paedomorphic populations of ambystomatids [20,26,28,52], salamandrids [17,24], and plethodontids [[27,53], this study] that inhabit relatively permanent aquatic systems, where surrounding terrestrial conditions often are harsh and not conducive for terrestrial salamanders. Arguably, temporary aquatic habitats strongly select for metamorphosis in salamanders with an aquatic larval stage [[50,51], this study]. Genetic crosses [54,55] and molecular genetic analyses [56-59] indicate that there are strong heritable components to the variations in developmental timing of closely related ambystomatid salamanders. Artificial selection studies on *Ambystoma talpoideum *demonstrated that pond drying can significantly increase the frequency of metamorphosis over very brief periods (only four generations) [51]. Toward the opposite extreme, obligately paedomorphic salamanders may arise from facultatively paedomorphic ancestors [15,20]. For example, based on genetic linkage mapping in captive versus wild populations of the axolotl (*Ambystoma mexicanum*), Voss and Shaffer [57] suggested that obligate paedomorphosis in captive populations may have resulted from artificial selection and/or bottlenecking when the colonies were established. The seemingly irreversible evolution of obligate paedomorphosis may be the result of either strong selection for paedomorphic "alleles", or loss-of-function mutations in genes associated with metamorphosis. Consequently, the removal of ecological, developmental or genetic constraints associated with metamorphosis or terrestriality may have allowed for the evolution of some of the most morphologically divergent salamanders. Aquifer-dwelling paedomorphic plethodontids such as *Eurycea rathbuni *and *Haideotriton wallacei *have extreme subterranean morphologies, and bear little resemblance to relatively closely related surface-dwelling species [43,44,48,60]. Indeed, the obligate paedomorphic salamanders of the families Sirenidae and Amphiumidae, each of which diverged from other salamanders by at least the Upper Cretaceous [61], have evolved to be similar in body form and ecology to "eel like" fishes and dipnoans.

### Life history evolution and reproductive isolation in plethodontids

More than two thirds (377 described species) of the world's salamanders are in the Family Plethodontidae [46], a group marked by extensive variation in ecology, life history, developmental timing and morphological innovation [53,62-64]. This diversity has been attributed, in part, to relatively low dispersal rates among populations [65], combined with divergence in courtship behaviours and evolution of associated pheromones [66-70]. These important aspects of plethodontid biology lead us to propose that reproductive isolation among populations exhibiting alternate developmental modes may occur more readily in plethodontids than other salamanders. The *Eurycea tynerensis *group will be a useful system for testing this hypothesis.

Morphological development and courtship behaviour are decoupled in the salamandrid *Triturus alpestris *[71,72], and paedomorphic and metamorphic individuals can court and reproduce despite having divergent morphologies. However, we suggest that the developmental shift from metamorphosis to paedomorphosis in plethodontids could have a profound impact on mate recognition. Almost all metamorphosing plethodontids exhibit terrestrial courtship, a strategy that is uncommon among the rest of the salamander families [66-70,73]. One diagnostic feature of adult metamorphosing plethodontids that is intimately associated with terrestrial courtship is the nasolabial groove, a tract on the surface of the skin that leads from the nostril to the margin of the upper lip [66-70,73]. To the best of our knowledge, the nasolabial groove only develops completely in fully metamorphosed individuals [[74-78], RMB pers obs, D. B. Wake pers com]. Therefore, we speculate that paedomorphic individuals may retain the larval aquatic communication system, while terrestrial metamorphs use other methods to find mates.

Spatial segregation in breeding ponds creates asymmetric reproductive isolation between sympatric morphs of the mole salamander, *Ambystoma talpoideum *[79]. Metamorphic males rarely encounter paedomorphic females, but no isolation is apparent between paedomorphic males and metamorphic females [79]. This asymmetry is considered to have little influence on sympatric speciation of the alternate phenotypes [79]. Little is known about the reproductive biology of *Eurycea tynerensis*, but there are notable differences between paedomorphic and metamorphosing populations [42,80]. For example, in many localities courting pairs of transformed individuals are commonly found at the edges of streams out of water, where it would be impossible for paedomorphs to reproduce [RMB pers obs]. Consequently, in addition to possible selection for single, distinct life history modes among populations, ecological, structural and behavioral differences could present barriers to communication between paedomorphs and metamorphs, promoting reproductive isolation in any areas where these two forms may meet. This could provide the basis for speciation.

## Conclusion

The geologically diverse Ozark Plateau is a unique landscape where distinctly different stream habitats occur in very close proximity. These habitat differences appear to strongly influence the development of *E. tynerensis*, either facultatively or by selection for alleles governing alternate developmental strategies, as most populations are unimodal in life history. The remarkably robust association between a relatively simple habitat feature such as streambed microstructure and developmental mode in salamanders of the *Eurycea tynerensis *group represents strong evidence for the role of microhabitat factors that may ultimately determine major changes in ontogeny, ecology, morphology and life history, and contribute to the process of speciation.

## Methods

### Sampling localities and specimens

*Eurycea tynerensis *were collected from throughout the Ozark Plateau of Arkansas, Missouri and Oklahoma by RMB and colleagues from August 2000 – August 2003. We selected 22 localities (11 paedomorphic and 11 metamorphic; Fig. 1; [see Additional File 1]) from throughout the geographic distribution, which represent the full spectrum of mitochondrial divergence within *E. tynerensis *[37]. Adult specimens from each of the 22 localities were collected for phylogenetic analyses. Salamanders were euthanised by submersion in a 10% solution of MS-222 according to IACUC protocols. Vouchers and tissues are in the University of Texas at Arlington (UTA) Amphibian and Reptile Diversity Research Center. Over a five year period (2000–2005) each of these localities was visited multiple times, and adult individuals observed at each locality were either all paedomorphic (sexually mature in the aquatic larval form) or all metamorphic (completely transformed). Sexual maturity of paedomorphs was determined by the presence of oviducal eggs in females and well-developed pigmented testes in males. At all the metamorphic localities, females guarding eggs and/or immature larvae were found on at least one visit, indicating that metamorphic *E. tynerensis *were breeding along these streams and were not simply transients.

### DNA isolation amplification and sequencing

DNA was isolated using DNeasy extraction kits (Qiagen). Portions of two mitochondrial genes, cytochrome *b *(*cob*, 1118 nucleotides) and NADH dehydrogenase subunit 4 (*nad4*, 700 nucleotides), were amplified via polymerase chain reaction (PCR). The primer pairs MVZ15 5'- GAA CTA ATG GCC CAC ACW WTA CGN AA -3' [81] and ETCR 5'- TTC TAA ACT ACA ACA GCA TC -3' [37] were used to amplify *cob*, and ND4F 5'- CAC CTA TGA CTA CCA AAA GCT CAT GTA GAA GC -3' [82] and EML1R 5'- CTT TCR TRT CTA GGG TCA CAG CCT AG -3' [37], were used for *nad4*. PCR products were electrophoresed on 1% agarose gels. Bands of the expected molecular weight were excised, purified and ligated into a plasmid vector using Topo-TA kits (Invitrogen). Plasmids were used to transform *E. coli *via heat shocking at 42°C. Transformed cells were plated on kanamycin/X-gal plates for blue/white selection. White colonies were picked and cultured overnight in Luria-Bertani broth. Plasmids were purified using a Qiagen Plasmid Miniprep kit and standard protocols, except that a 1:10 dilution of EB buffer:/water was used for the final elution. Approximately 4 μl of plasmid was dried for sequencing. SequiTherm Excel ™ sequencing kits (Epicentre Technologies) and infrared labeled primers were used for sequencing reactions. Plasmids from at least two different clones of each fragment, for each specimen, were bi-directionally sequenced on a LiCor 4200 L long-read, dual-laser sequencer. Both strands were sequenced completely for each sample.

### Sequence alignment and phylogenetic analyses

We used Sequencher ™ 3.1 (Gene Codes Corp.) to align and edit sequences. No indels were observed, and alignment was unambiguous. Both gene fragments were translated in MacClade [83], and no stop codons were found. Sequences were deposited in GenBank [see Additional File 1]. A Nexus file containing a concatenated alignment of both genes was analyzed using Bayesian methodology. *Eurycea multiplicata *and *E. spelaea*, two species closely related to *E. tynerensis *[37,47], were used as outgroups to root the trees. ModelTest v. 3.06 [84] was used to calculate the most appropriate model of nucleotide substitution. ModelTest selected the same model (HKY + I + Γ) for both genes. For *cob*, transition/transversion (Ti/Tv) ratio = 8.4750, proportion of invariable sites (I) = 0.4856, gamma distribution shape parameter (Γ) = 0.8822, and mean base frequencies were A = 0.3078; C = 0.2557; G = 0.1341; T = 0.3024. For *nad4*, Ti/Tv ratio = 11.2622, I = 0.3496, Γ = 0.3835, and mean base frequencies were A = 0.3404; C = 0.2768; G = 0.1179; T = 0.2649.

The data were partitioned by gene (*cob *and *nad4*) for Bayesian analyses (all partitions unlinked) implemented in MrBayes 3.0 [85]. Analyses were run with four chains and uniform priors for 5.05 million generations (with the first 50,000 discarded as burn-in; stationarity of likelihoods achieved before this point). The resulting 50% majority-rule consensus of the 50,000 post burn-in trees, sampled every 100 generations, was computed in PAUP*v 4.0b10 [86]. Bayesian branch lengths for the combined data set were averaged across trees in MrBayes 3.0 [85]. Unweighted maximum parsimony analysis and nonparametric bootstrapping were also performed using PAUP*. The resulting tree was congruent in most respects with the Bayesian tree, with strong support for the same nodes that were highly supported in the Bayesian analysis. Phylogenetic independent contrasts (see below) using topologies and branch lengths from both methods of analyses yielded nearly identical results. Given the high degree of congruence between the results of both analyses, we only show the results from Bayesian analyses.

### Substrate sampling and quantification of size and type

Three stream substrate samples were collected from each of the 22 localities. The first substrate sample was collected immediately adjacent to where the first *E. tynerensis *at each locality was found during the sampling period. The other two samples were randomly selected from one to six meters up and downstream from where the first substrate sample was collected. The number of meters (1 to 6) was decided by rolling a six-sided die. Substrate samples were returned to the University of Texas at Arlington and dried completely under incandescent lamps. Dried substrate samples were sorted by size using a cylinder of eight hierarchically stacked screen sieves. Substrate samples were poured into the top of the cylinder, which was then capped and fixed to an orbit shaker (Lab Line Instruments Inc.) and shaken at 250 rpm for five minutes. The sieves separated the substrate into eight size classes (0 to < 0.25, 0.25 to < 0.5, 0.5 to < 2, 2 to < 4, 4 to < 6, 6 to < 12, 12 to < 25, & ≥ 25 mm) based on mesh diameter. The total volume of substrate (in ml) in each category was measured by water displacement in graduated cylinders. The three substrate samples collected at each locality were combined on the basis of their extreme similarity.

### Independent contrasts

Testing for correlations between organismal traits and their environments is one of the many uses of phylogenetic comparative methods [38-40]. We used phylogenetic independent contrasts (PIC) [38] calculated in COMPARE version 4.6 [87] to account for possible phylogenetic non-independence of our data, when testing for a correlation between stream substrate parameters (size, degree of sorting and type) and life history mode. We based our comparative analysis on the Bayesian phylogeny and branch lengths, although results based on the maximum parsimony tree were almost identical (data not shown). Our Bayesian topology had two trichotomies. However, COMPARE cannot handle polytomies [87]. To resolve these ambiguities we arbitrarily placed P8 as sister to the clade containing M8, P9, M7, P7, P10 and P11, and we made P5 and P6 a clade. We assigned extremely short (0.000001 substitutions/site) lengths to internal branches subtending these nodes [87].

Life history of salamanders at each locality was scored as a categorical variable: paedomorphic (0) and metamorphic (1). PIC of life history mode was calculated against four continuous substrate parameters (the proportion of sand and granule, the proportion of small/medium gravel, the number of substrate size classes, and the proportion of chert) for the 22 localities. Standard errors of all variables were set to zero. Using SAS v. 8.2 (SAS institute Inc.), 1000 Markov Chain Monte Carlo (MCMC) random iterations were used to create a random distribution of r^2 ^values based on the original data. To assess significance (p < 0.05), our observed r^2 ^for each regression was compared to this distribution to test the relationship between life-history mode and a given substrate parameter. We also found streams containing large gravel to be positively correlated with paedomorphosis, but we do not formally test this because it is simply an "inverse" test of sand and granule association with developmental mode.

The original application of phylogenetic comparative methods such as independent contrasts was for interspecific comparisons, although intraspecific studies have highlighted the utility of these methods within species [88-90]. Recently, Niewiaroski and others [91] questioned the necessity of applying phylogenetic comparative methods below the species level. They pointed out the possibility of making Type I errors (overlooking significant correlations) by using such a conservative method at shallow levels of divergence, but noted that more studies at this scale are warranted. Even though phylogenetic comparative methods may be statistically conservative, we implement them here to be certain that our results are not biased by common ancestry.

## Abbreviations

*cob*, mitochondrial gene cytochrome b; M, metamorphic; *nad4*, mitochondrial gene NADH dehydrogenase subunit 4; P, paedomorphic; PICs, phylogenetic independent contrasts; UTA, University of Texas at Arlington.

## Authors' contributions

RMB conceived the experiment, collected and analyzed the data, and primarily prepared the manuscript. The work was conducted in the laboratory of PTC, who contributed to the study design, analyses and manuscript preparation.

## Supplementary Material

Additional File 1 Locality information, and museum and Genbank accession numbers for the 22 populations of *E. tynerensis *and outgroups. Click here for file
