# Supplementary material for: Streambed microstructure predicts evolution of development and life history mode in the plethodontid salamander Eurycea tynerensis
Source: BMC Biol. 2006 Mar 2;4:6. doi: 10.1186/1741-7007-4-6 (PMC1413558; doi:10.1186/1741-7007-4-6)
Supplement: Additional File 1 — Locality information, and museum and Genbank accession numbers for the 22 populations of E. tynerensis and outgroups. [file 1741-7007-4-6-S1.pdf]

**Additional File 1. Locality information and representative mitochondrial DNA sequences.**

| Tree code/ map number | Locality | Museum Accession Number | Genbank Accession *nad4* | Genbank Accession *cob* |
|---|---|---|---|---|
| M1 | AR: Van Buren Co., Weaver Creek | UTA 56374 | AY528272 | AY528347 |
| M2 | AR: Clerburne Co., stream near Ida | UTA 57119 | DQ358999 | DQ359006 |
| M3 | AR: Van Buren Co., Bradley Brook, near Clinton | UTA 57120 | DQ359000 | DQ359007 |
| M4 | AR: Van Buren Co., stream near Cardon Cr. | UTA 57118 | DQ359001 | DQ359008 |
| M5 | AR: White Co., Little Creek | UTA 56375 | AY528273 | AY528348 |
| M6 | AR: Marion Co., Gray Spring | UTA 56381 | AY528279 | AY528354 |
| M7 | AR: Newton Co., Low Gap Springs | UTA 56389 | AY528288 | AY528363 |
| M8 | AR: Benton Co., Ashmore Creek | UTA 56387 | AY528286 | AY528361 |
| M9 | OK: Sequoyah Co., Tin Cup Creek | UTA 56402 | AY528293 | AY528368 |
| M10 | OK: Sequoyah Co., 10 mi NE of Gore | UTA 56398 | AY528291 | AY528366 |
| M11 | OK: Sequoyah Co., near Cookson | UTA 56399 | AY528292 | AY528367 |
| P1 | OK: Cherokee Co., Rock Creek | UTA 53860 | AY528299 | AY528374 |
| P2 | OK: Cherokee Co., Tyner Creek | UTA 57121 | DQ359002 | DQ359009 |
| P3 | OK: Cherokee Co., Peavine Creek | UTA 56405 | AY528302 | AY528377 |
| P4 | OK: Cherokee Co., Cave spring, near Peggs | UTA 57161 | DQ359003 | DQ359010 |
| P5 | OK: Delaware Co., Spring near Colcord | UTA 56410 | AY528305 | AY528380 |
| P6 | AR: Benton Co., Spavinaw Creek, at Rt 43. | no voucher | DQ359004 | DQ359011 |
| P7 | MO: McDonald Co., Mill Creek | UTA 56392 | AY528306 | AY528381 |
| P8 | MO: McDonald Co., Mike's Creek | UTA 56393 | AY528307 | AY528382 |
| P9 | MO: McDonald Co., Big Sugar Creek | UTA 56394 | AY528308 | AY528383 |
| P10 | MO: Stone Co., Pine Run | UTA 56395 | AY528309 | AY528384 |
| P11 | MO: Christian Co., Buseik S.F. | no voucher | DQ359005 | DQ359012 |
| *E. spelaea* | MO: Barry Co., Rockhouse Cave | UTA 56364 | AY528318 | AY528393 |
| *E. multiplicata* | OK: Choctow Co., near Ft. Towson | UTA 56367 | AY528255 | AY528330 |
